# From validity to clinical utility: the influence of circulating tumor DNA on melanoma patient management in a real‐world setting

**DOI:** 10.1002/1878-0261.12373

**Published:** 2018-09-08

**Authors:** Steven P. Rowe, Brandon Luber, Monique Makell, Patricia Brothers, JoAnn Santmyer, Megan D. Schollenberger, Hannah Quinn, Daniel L. Edelstein, Frederick S. Jones, Karen B. Bleich, William H. Sharfman, Evan J. Lipson

**Affiliations:** ^1^ The Russell H. Morgan Department of Radiology and Radiological Science Johns Hopkins University School of Medicine Baltimore MD USA; ^2^ Division of Biostatistics and Bioinformatics Department of Oncology Sidney Kimmel Comprehensive Cancer Center Johns Hopkins University School of Medicine Baltimore MD USA; ^3^ Department of Oncology Sidney Kimmel Comprehensive Cancer Center Johns Hopkins University School of Medicine Baltimore MD USA; ^4^ Sysmex Inostics Inc. Mundelein IL USA

**Keywords:** circulating tumor DNA, ctDNA, melanoma

## Abstract

Melanoma currently lacks a reliable blood‐based biomarker of disease activity, although circulating tumor DNA (ctDNA) may fill this role. We investigated the clinical utility (i.e., impact on clinical outcomes and interpretation of radiographic data) of measuring ctDNA in patients with metastatic or high‐risk resected melanoma. Patients were prospectively accrued into ≥ 1 of three cohorts, as follows. Cohort A: patients with radiographically measurable metastatic melanoma who underwent comparison of ctDNA measured by a BEAMing digital PCR assay to tissue mutational status and total tumor burden; when appropriate, determinations about initiation of targeted therapy were based on ctDNA data. Cohorts B and C: patients with BRAF‐ or NRAS‐mutant melanoma who had either undergone surgical resection of high‐risk disease (cohort B) or were receiving or had received medical therapy for advanced disease (cohort C). Patients were followed longitudinally with serial ctDNA measurements with contemporaneous radiographic imaging to ascertain times to detection of disease activity and progressive disease, respectively. The sensitivity and specificity of the ctDNA assay were 86.8% and 100%, respectively. Higher tumor burden and visceral metastases were found to be associated with detectable ctDNA. In two patients in cohort A, ctDNA test results revealed a targetable mutation where tumor testing had not; both patients experienced a partial response to targeted therapy. In four of 30 patients with advanced melanoma, ctDNA assessments indicated evidence of melanoma activity that predicted radiographic evidence of disease progression by 8, 14, 25, and 38 weeks, respectively. CtDNA was detectable in three of these four patients coincident with radiographic evaluations that alone were interpreted as showing no evidence of neoplastic disease. Our findings provide evidence for the clinical utility of integrating ctDNA data in managing patients with melanoma in a real‐world setting.

**Abbreviations**
BEAMing: beads, emulsions, amplification, magnetics
cfDNA: circulating free DNA
ctDNA: circulating tumor DNA
EDA: evidence of disease activity

## 1 Introduction

Circulating tumor DNA (ctDNA) has recently emerged as a candidate blood‐based biomarker of melanoma activity. Our group and others have demonstrated the analytical and clinical validity of ctDNA testing as a method for monitoring tumor burden and predicting outcomes in patients receiving immune checkpoint blockade therapy (Bettegowda *et al*., 2014; Lee *et al*., 2017; Lipson *et al*., 2014). Intrapatient trends observed among serial ctDNA measurements were shown to differentiate progressive disease from an immune‐related tumor response (Lee *et al*., 2018a,b). In addition, patients with advanced melanoma receiving inhibitors of the mitogen‐activated protein kinase (MAPK) pathway showed marked increases in ctDNA that preceded the radiographic appearance of progressive disease, suggesting genomic changes linked to acquired drug resistance (Gray *et al*., 2015; Wong *et al*., 2017). Among patients with high‐risk resected melanoma, ctDNA levels have been shown to predict disease relapse (Lee *et al*., 2018a,b). Taken together, these findings suggest that incorporation of ctDNA as a melanoma biomarker into the clinical setting could improve the current standard of care by providing early information about neoplastic growth and complementing radiographic imaging as an accurate gauge of treatment efficacy.

In this study, we explored the clinical utility of integrating ctDNA data (i.e., impacts on patient management and outcomes) for patients with melanoma in a real‐world setting. Among patients with metastatic melanoma, we first evaluated the concordance of somatic mutation testing results obtained in tumor tissue using standard‐of‐care analyses with those detected in circulating free DNA (cfDNA) in patient plasma using a digital PCR‐based BEAMing (beads, emulsions, amplification, magnetics) assay. We then assessed ctDNA levels among patients with a wide range of melanoma tumor burdens involving various organs to better understand correlations between disease volume, tumor location, and the performance of the BEAMing assay. In appropriate clinical circumstances, we allowed ctDNA testing results to inform treatment decisions and analyzed resultant outcomes.

Further, in order to assess how ctDNA testing data might impact interpretations of standard‐of‐care radiographic assessments, we performed serial measurements of ctDNA levels in patients and compared these data with results from concurrent imaging evaluations. Imaging was being performed in order to assess response to cancer therapy or monitor for melanoma relapse in patients who had undergone surgical resection of disease. The overarching goal of these investigations was to test the hypothesis that plasma‐based ctDNA data complements radiographic imaging data, providing actionable information about disease activity and informing clinical decision‐making in this patient population.

## 2 Materials and methods

### 2.1 Patients

After approval from the Johns Hopkins University Institutional Review Board, patients with melanoma undergoing evaluation and/or treatment signed written informed consent. The study methodologies conformed to the standards set by the Declaration of Helsinki. Archived formalin‐fixed, paraffin‐embedded tumor specimens from each patient were analyzed for common, recurrent somatic mutations, in the oncogenes *BRAF* and *NRAS* using standard techniques (e.g., next‐generation sequencing or pyrosequencing). Patients were accrued into one or more of three cohorts as follows:

Cohort A: Patients with radiographically measurable metastatic melanoma, regardless of tumor mutation status, underwent measurement of ctDNA at a single time point coincident with imaging used to calculate tumor burden. All visible sites of metastatic disease were measured using contemporaneous diagnostic computed tomography (CT) or magnetic resonance imaging (longest diameter in the axial plane), and the organs in which tumors were present were recorded. Rates of concordance between mutations detected in tumor tissue and those detected in plasma were calculated. Total radiographic tumor burden measurements [sum of longest diameters (SLD)] were compared with ctDNA levels for each patient. The following somatic mutations were measured in plasma: [*BRAF* 1799T>A (V600E) or 1798_1799delGTinsAA (V600K); *NRAS* 181C>A (Q61K), 182A>G (Q61R), 183A>T or 182A>T (Q61L), or 183A>C (Q61H)]. Using tumor tissue mutation testing results as the gold standard and a lower limit of detection (LoD) of 0.03% mutant alleles/wild‐type DNA in plasma, receiver operating characteristic (ROC) analysis was performed to calculate maximum sensitivity and specificity, and area under the curve (AUC) with 95% bootstrapped confidence intervals, computed using 2000 bootstrap replicates. Statistical analyses were performed using the R statistical package (version 3.4.0) (The R Foundation for Statistical Computing, Vienna, Austria) to explore the clinical correlation of the ctDNA assay to the anatomical location of metastases and total tumor burden necessary for ctDNA to be reliably detectable.
Cohort B: Patients with surgically resected high‐risk melanoma (AJCC stage IIB–IV) whose tumor tissue analysis revealed any of the aforementioned seven hotspot mutations in *BRAF* and *NRAS* underwent serial plasma collections coincident with standard‐of‐care radiographic studies so as to provide parallel ctDNA and radiographic data in order to investigate an integrated approach to monitoring for melanoma recurrence. Information from clinical imaging reports was collated, and the images were re‐reviewed by a study oncologist (EJL) and a study radiologist (SPR). Radiographic evidence of locoregional and distant disease progression was compared to ctDNA‐based evidence of disease activity (EDA), defined as ≥ 0.03% mutant alleles/wild‐type DNA detected in plasma (lower LoD of the ctDNA detection assay, described below).
Cohort C: Patients with unresectable or metastatic melanoma whose tumor tissue analysis revealed any of the aforementioned seven hotspot somatic mutations in *BRAF* and *NRAS* and who were receiving or had received systemic cancer therapy underwent serial plasma collections coincident with standard‐of‐care radiographic studies performed to assess response to treatment. Metastases were measured at each imaging time point, and radiographic PFS was calculated from time of first radiographic/ctDNA assessment to the date of radiographic progression or death. Time to radiographic disease progression per RECIST 1.1 criteria (Eisenhauer *et al*., 2009) calculated from first on‐trial assessment was compared with time to EDA in ctDNA. Longitudinal intrapatient ctDNA trends were interrogated for actionable information about disease status (e.g., ctDNA‐based EDA seen in the setting of a complete radiographic response) that would inform clinical decision‐making in this patient population.

### 2.2 Patient sample acquisition and plasma mutation testing using BEAMing

For all patient cohorts, 10–20 mL of whole blood was prospectively collected in Cell‐Free DNA blood collection tubes (Streck La Vista, NE) at each time point. Blood samples were transported in temperature‐controlled shipment containers designed and validated to maintain sample core temperatures between 18 and 30 °C (Diaz *et al.,* 2016). Plasma was prepared from collected blood within 48 h according to CLIA‐validated procedures for ctDNA testing (Sysmex Inostics, Inc., Baltimore, MD, USA).

Preparation of plasma included a two‐step centrifugation with blood initially centrifuged for 10 min at 1600 ***g*** at room temperature. Supernatant was then collected, avoiding the buffy coat, and centrifuged again for 10 min at room temperature at 6000 ***g*** to remove remaining cells. Plasma supernatant was then transferred into a 2‐mL cryogenic tube and stored at −80 °C until analysis. DNA was purified from 2 mL of plasma from each blood sample and thawed at room temperature for 15 min prior to ctDNA isolation. Purification of DNA from plasma was performed using the QIAamp DSP DNA purification kit (Qiagen, Venlo, Netherlands) according to the manufacturer's instructions. The total amount of amplifiable human genomic DNA purified from plasma samples was quantified using a modified version of human long interspersed element 1 (LINE‐1) real‐time PCR assay and reported as genome equivalents (GE; Diehl *et al*., 2008; Rago *et al*., 2007). BEAMing analysis was performed on the total DNA content purified from 2 mL of plasma. Plasma samples with < 500 total GE were deemed insufficient for mutational analysis.

All plasma samples were analyzed by BEAMing (beads, emulsions, amplification, magnetics) as validated in Sysmex Inostics’ CLIA‐certified laboratory for two mutations in BRAF [c.1799T>A (V600E); c.1798_1799delGTinsAA (V600K)] and five mutations in NRAS [c.181C>A (Q61K); c.182A>G (Q61R); c.183A>T, c.182A>T (Q61L); c.183A>C, (Q61H)]. BEAMing utilizes emulsion digital PCR performed on magnetic beads to amplify single DNA molecules (Diehl *et al*., 2006). Preamplification was performed with a first amplification of multiple loci in a multiplex PCR reaction, followed by a second preamplification with nested primers for individual amplicons. Subsequently, emulsion PCR was performed with amplification on the surface of magnetic beads in oil–water emulsions subjected to thermal cycling. Individual beads were then hybridized to allele‐specific fluorescently labeled probes complementary to the mutant and wild‐type DNA sequences. Finally, the bead population was analyzed by flow cytometry to count and sort wild‐type and mutant beads. The result was reported as both the fractional abundance of mutant DNA alleles relative to wild‐type DNA alleles in a plasma sample and the absolute number of mutant alleles. To generate the ratio of mutant to wild‐type DNA alleles [mutant allelic fraction (MAF)], a maximum of 1 × 10^6^ beads were interrogated in each BEAMing analysis.

Plasma samples were determined to be positive for a given mutation in BRAF or NRAS if the mutation was detected above 0.03% MAF (Sysmex Inostics, Inc., Internal Validation). This mutation cutoff threshold was set to ensure that the LoDs for each amplicon in the BRAF and NRAS assay were set well above background signals or limits of blank (LoBs) for each analyte to be detected in clinical samples. LoDs were determined by probit regression analyses by spiking wild‐type (normal sequence BRAF/NRAS) plasma with each BRAF/NRAS amplicon at varying inputs of mutant DNA in the presence of the same amount of total DNA. Background signal (LoB) was determined from DNA prepared from wild‐type plasma samples lacking BRAF or NRAS mutations at low, medium, and high concentrations of genomic DNA and unambiguously determined to be wild‐type across this series. Based on the results of these experiments, the cutoff of 0.03% mutant alleles/wild‐type DNA was determined as appropriate to obtain 95% probability confidence interval of reporting a result of ‘mutation detected’. In certain cases where plasma mutations were detected, mutant molecules per milliliter of plasma (MM/mL) were reported alongside of MAF to examine both values with respect to overall tumor burden. Mutant copy numbers were calculated by multiplying the percentage of mutant beads by the number of GE.

### 2.3 Image analysis

All imaging studies were viewed on our institution's clinical picture archiving and communication system (CareStream, Rochester, NY, USA). Standard‐of‐care imaging interpretation was performed by board‐certified clinical radiologists who did not have access to ctDNA measurements. Images were then re‐reviewed by a board‐certified study radiologist (SPR) in an unblinded fashion (i.e., informed by ctDNA results). Incongruities between interpretations were noted. Determinations regarding response to therapy were performed in collaboration with a board‐certified medical oncologist (EJL) according to Response Evaluation Criteria in Solid Tumors (RECIST) version 1.1 as well as immune RECIST (iRECIST) response criteria (Eisenhauer *et al*., 2009; Seymour *et al*., 2017).

## 3 Results

Patients were prospectively enrolled into ≥ 1 of three cohorts to examine the utility of plasma ctDNA analysis in different clinical scenarios. These cohorts include the following attributes: Cohort A represents patients with radiographically measurable metastatic melanoma who underwent a comparison of plasma ctDNA mutation results versus results determined by tissue mutation testing with correlation to overall tumor burden. Cohorts B and C comprised patients with BRAF‐ or NRAS‐mutant melanoma who had either undergone surgical resection of high‐risk disease (cohort B) or were receiving or had received medical therapy for advanced disease (cohort C). Overall, 260 plasma mutation results with BEAMing were generated for patients across all three cohorts. The median LINE‐1 concentration inputted into each BEAMing assay was 4953 GE (interquartile range 3613–7917 GE) per 2 mL of plasma.

### 3.1 Cohort A

Between July 2015 and June 2017, 60 patients with radiographically measurable metastatic melanoma were accrued. Patient demographic and tumor mutation data are shown in Table 1. Average duration from tissue acquisition to plasma acquisition was 16.4 months (range −0.2 to 85.2). Twenty‐three of 60 (38%) patients had received or were receiving systemic treatment prior to plasma acquisition. Tumor tissue testing revealed one of the seven mutations of interest (BRAF 1799T>A, 1798_1799delGTinsAA; NRAS 181C>A, 182A>G, 183A>T, 182A>T, 183A>C) in 38/60 (63%) patient samples. In 33 of those 38 patients (86.8%), mutations identified in circulation exactly matched the mutations found in tumor specimens. In the remaining five patients, all of whom were naïve to systemic therapy, no mutation was detected in circulation using a lower LoD of 0.03% mutant alleles/wild‐type DNA, as described above. The sensitivity of the assay used in this real‐world setting (86.8%, 95% CI, 72–96) approximates sensitivity estimates from previous reports (Tables 1 and 2; Bettegowda *et al*., 2014, Santiago‐Walker *et al*., 2016).

**Table 1:** Patient demographic and tumor mutation data

	Cohort A^a^; *n* = 60	Cohort B^b^; *n* = 31	Cohort C^c^; *n* = 36
Age (years; median, range)	60.7 (24–87.7)	51.9 (22.4–73.1)	58.6 (29.3–86.1)
Gender, *n* (%)			
Male	38 (63)	16 (52)	14 (39)
Female	22 (37)	15 (48)	22 (61)

Mutation detected	Tumor‐based	Plasma‐based (ctDNA)	Tumor‐based		Tumor‐based	
BRAF V600E	21	17	19		23	
BRAF V600K	6	5	2		2	
NRAS Q61H	0	0	1		0	
NRAS Q61K	2	2	4		1	
NRAS Q61L	1	1	2		0	
NRAS Q61R	8	8	3		9	
Wild‐type	17	N/A	N/A		N/A	
Nonevaluable	5^d^	See footnote^d^	0		1^e^	
Stage at study entry (AJCC version 7)	IV M1a	1	IIB	1	IV M1a	6
	IV M1b	6	IIIA	8	IV M1b	6
	IV M1c	53	IIIB	11	IV M1c	24
			IIIC	10		
			IV	1		

^a^Patients with radiographically measurable metastatic melanoma regardless of tumor mutation. ^b^Patients with surgically resected high‐risk melanoma (AJCC stage IIB–IIIC) whose tumor tissue analysis revealed any of the following somatic mutations [*BRAF* 1799T>A (V600E) or 1798_1799delGTinsAA (V600K); *NRAS* 181C>A (Q61K), 182A>G (Q61R), 183A>T or 182A>T (Q61L), or 183A>C (Q61H)]. ^c^Patients with unresectable or metastatic melanoma whose tumor tissue analysis revealed any of the above‐referenced somatic mutations and who were receiving or had received systemic anti‐neoplastic therapy. ^d^Tumor tissue from five patients was of insufficient quantity or quality to perform mutation testing and were, therefore, unevaluable for the concordance portion of the study. Of those five patients, one patient was found to have a circulating BRAF V600E mutation. That patient experienced a partial response to dabrafenib and trametinib (BRAF and MEK inhibitors, respectively), ongoing at 12 months. ^e^Tumor tissue from one patient was found to contain a BRAF V600 mutation (COBAS assay); however, information about the specific mutation was not available.

**Table 2:** Concordance between mutations detected in plasma (ctDNA) and tissue among 55 evaluable patients in cohort A

	Mutation detected in tumor tissue		Total
	(+)	(−)	
Mutation detected in plasma (ctDNA)			
(+)	33	0	33
(−)	5	17	22
Total	38	17	55

Positive percent agreement = 86.8% (95% CI 72–96); Negative percent agreement = 100% (95% CI 78–100); Overall percent agreement = 90.9% (95% CI 80–97).

One patient whose primary melanoma tumor was reported to be wild‐type for BRAF (COBAS Real‐Time Polymerase Chain Reaction performed in a CLIA‐approved Molecular Diagnostics Laboratory) was found to have a circulating BRAF V600E mutation. Notably, this finding allowed for treatment of the patient with dabrafenib (BRAF inhibitor) and trametinib (MEK inhibitor), leading to a partial response (RECIST 1.1). Subsequent next‐generation sequencing of genetic material from metastatic tumor cells from a malignant pleural effusion revealed a BRAF V600E mutation, corroborating the ctDNA testing result. This patient was, therefore, included as a concordant result.

Seventeen of the 60 tumor samples (28%) were either wild‐type for the above‐referenced genes (13/17, 76%) or harbored mutations that were not assessed in plasma using the BEAMing methodology described above (one each with NRAS G12R, NRAS G12C, PIK3CA N319K, and BRAF R603*). No circulating BRAF V600E/K or NRAS Q61H/K/L/R mutation was detected in any of those 17 patients. Overall, specificity of the assay was 100% (95% CI, 80.5–100), which approximates previously reported specificity estimates (Ascierto *et al*., 2013).

Tumor tissue from five patients was of insufficient quantity or quality to perform mutation testing; therefore, these patients were unevaluable for the concordance portion of the study. However, among those five patients, one was found to have a circulating BRAF V600E mutation. This patient, whose medical history includes severe rheumatoid arthritis (a relative contraindication to administration of immune checkpoint blocker), experienced a partial response to dabrafenib and trametinib, ongoing at 12 months.

Thirty‐eight patients in cohort A had radiographically measurable metastatic disease and were evaluable for disease burden estimation. Average total tumor burden was 423 mm (range 7–4278). Average MAF was 7.1% (range 0.03–32.8). ROC analysis demonstrated that optimum sensitivity and specificity for tumor detection occurred at 35.5 mm of total tumor diameter measured in the axial plane [sensitivity 88.2%, specificity 95.2%, AUC 97% (95% CI, 92–100)]. CtDNA was detectable (average MAF = 7.4%, range 0.03–32.8; median mutant molecules/mL = 26.24, interquartile range of 8.42–1162.0) in 22 of 27 (81%) with BRAF mutations, in whom the average tumor burden was 379 mm (range 7–4278). CtDNA was detectable (average MAF = 6.5%, range 0.06–31.2; median mutant molecules/mL = 58.30, interquartile range of 6.13–373.70) in all 11 patients (11/11, 100%) with NRAS mutations, in whom the average tumor burden was 550 mm (range 20–2639).

**Figure 1:**
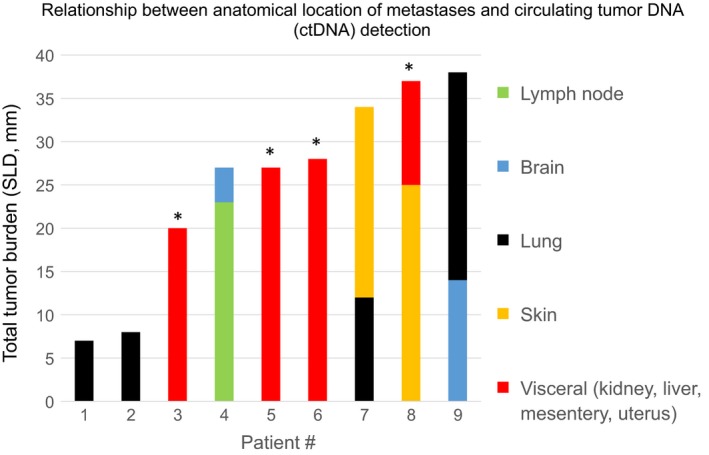
Relationship between anatomical location of melanoma metastases and circulating tumor DNA (ctDNA) detection. The anatomical distribution of disease among nine treatment‐naïve patients with metastatic melanoma with < 40 mm of total tumor burden appeared to have an impact on the likelihood of ctDNA detection. Asterisks indicate the four patients in whom ctDNA was detectable, all of whom had visceral metastases. CtDNA was not detectable in the remaining five patients, whose metastases were limited to lymph nodes, brain, lung, and/or skin. SLD, sum of longest diameters.

**Figure 2:**
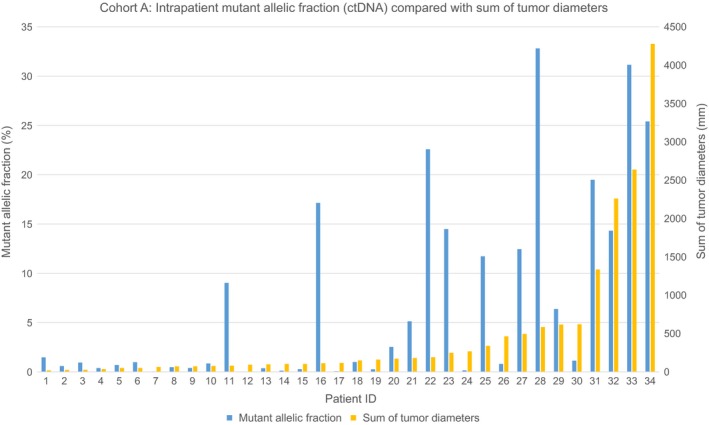
Intrapatient ctDNA MAF compared with sum of tumor diameters (SLD) among all 34 patients with metastatic melanoma in cohort A. The Pearson product moment correlation coefficient between MAF and SLD was *r* = 0.64, suggesting a moderate‐to‐strong linear relationship between the two metrics.

All 24 patients with > 50 mm of total tumor burden had measurable ctDNA. Of these 24 patients, 20 (83%) had visceral metastases. The anatomical distribution of disease among the nine patients in cohort A with < 40 mm of total tumor burden (SLD) appeared to have an impact on the likelihood of ctDNA detection (Fig. 1). Of those nine patients, ctDNA was detected in four, all of whom had visceral metastases. Plasma‐based tumor‐derived DNA was not detectable in the remaining five patients (all treatment‐naïve), whose metastases were limited to lymph nodes, brain, lung, and/or skin. Similar observations have been previously described in metastatic lung and colorectal cancer patients with isolated lung metastases (Garcia Foncillas *et al*., 2017; Karlovich *et al*., 2016). For all 34 patients with detectable ctDNA (Fig. 2), the Pearson product moment correlation coefficient between MAF and SLD was *r* = 0.64, suggesting a moderate‐to‐strong linear relationship between the two metrics.

### 3.2 Cohort B

Between August 2015 and September 2016, cohort B accrued 31 patients with surgically resected high‐risk melanoma (stage IIB–IV, American Joint Committee on Cancer (AJCC) version 7) containing one of the seven mutations of interest described above. Patient demographic, disease stage, and tumor mutation data for patients in cohort B are shown in Table 1. All patients had no radiographic evidence of disease prior to enrollment. Ten patients of 31 (32%) received adjuvant medical therapy after surgical resection, including interferon (2/31, 6%), ipilimumab (2/31, 6%), dabrafenib plus trametinib or two placebos (5/31, 16%; Long *et al*., 2017), or an experimental melanoma vaccine‐based regimen (1/31, 3%; NCT02126579).

One patient who was lost to follow‐up and another who was diagnosed with lymphoma were unevaluable. Among the remaining 29 evaluable patients, radiographic imaging and corresponding ctDNA fraction determinations were performed at ~8‐ to 12‐week intervals. The average number of assessment time points was 2.8 (range 1–5). The average length of follow‐up was 8.7 months (range 0–16). Five patients (two BRAF V600E, one BRAF V600K, two NRAS Q61R) of 29 (17%) were found through clinical examination or imaging to have recurrent melanoma during the study period. Three patients developed biopsy‐proven locoregional recurrences [two in lymph nodes (1 and 0.9 cm) and one in subcutaneous tissue (1.6 cm)], none of which was detected by the ctDNA assay. One of these patients and two others subsequently developed distant metastases in lung (single 7‐mm lesion, subsequently resected revealing BRAF V600E melanoma, undetected by ctDNA), liver (BRAF V600E, detected), and kidney (NRAS Q61R, detected). Radiographic and ctDNA results are summarized in Table S1.

### 3.3 Cohort C

Between August 2015 and December 2016, cohort C accrued 36 patients who had received or were, at the time of study enrollment, receiving medical therapy for locally advanced unresectable or metastatic melanoma containing one of the seven mutations of interest described above. Patient demographic, disease stage, and tumor mutation data are shown in Table 1. Radiographic imaging and corresponding ctDNA mutant fraction determinations (summarized in Table S2) were performed at ~8‐ to 12‐week intervals. Of 30 evaluable patients, the average number of assessment time points was 3.7 (range 2–6). Average length of follow‐up was 8.4 months (range 2.3–17.9). Therapies included one or more of the following: immune checkpoint inhibition (e.g., anti‐PD‐1, anti‐CTLA‐4; *n* = 24), targeted therapy (e.g., dabrafenib, trametinib; *n* = 8), radiotherapy (*n* = 6), high‐dose interleukin 2 (IL‐2; *n* = 1), and temozolomide (*n* = 1).

Seventeen patients (57%) experienced a partial or complete radiographic response to therapy. No EDA was detected in ctDNA (average number of time points = 3.6, range 2–5) in any of those patients after initial on‐treatment radiographic disease assessment.

One patient (3%) developed a partial radiographic response to ipilimumab + nivolumab. EDA was repeatedly seen in four ctDNA measurements over ~6 months. A CT scan performed 10 weeks after his last on‐trial assessment revealed progressive disease. One patient experienced a partial response (PR) but had to switch therapies due to toxicity; her ctDNA remained persistently detectable. Three patients experienced stable disease over 10, 20, and 31 weeks, respectively; two had persistent EDA in ctDNA, one became undetectable.

**Figure 3:**
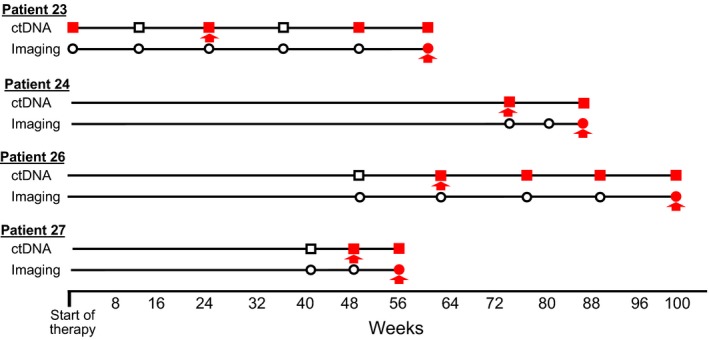
Graphical representation of the time course to progression of the four patients from cohort C in whom ctDNA detection preceded evidence of disease progression on radiographic imaging. Open squares indicate undetectable ctDNA levels, and solid red squares represent detectable ctDNA. Open circles signify nonprogressive disease imaging findings (e.g., baseline, stable disease, partial response, complete response); solid red circles indicate radiographic evidence of disease progression. The distance between each pair of red arrows denotes the time from initial detection of ctDNA‐based EDA (after baseline) until disease progression on radiographic imaging. Detectable ctDNA preceded evidence of radiographic disease progression by an average of 21 weeks (range 8–38) in this patient group.

**Figure 4:**
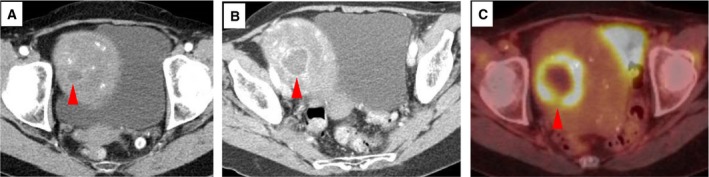
EDA detected in circulating tumor DNA (ctDNA) predicts radiographic melanoma progression. (A) Representative image from a contrast‐enhanced CT scan obtained at study entry of a 68‐year‐old female receiving pembrolizumab (anti‐PD‐1) for metastatic melanoma. Although ctDNA was detectable at this time point (and remained so thereafter), this examination was described as demonstrating no evidence of disease. However, a subtle, rim‐enhancing, centrally necrotic 1.5 cm mass (red arrowhead) is present within a large uterine fibroid. (B) Contrast‐enhanced CT image at 3 months demonstrating an interval increase in the intrafibroid lesion, now 3 cm in diameter. The CT images were again described as demonstrating no evidence of melanoma. (C) FDG‐PET/CT image at 5 months demonstrating a 5‐cm necrotic hypermetabolic lesion within a fibroid uterus. Subsequent biopsy of this mass proved the presence of metastatic melanoma.

The remaining eight patients developed radiographic PD over the course of the study. In four of those patients, PD was coincident with detection of EDA in ctDNA. In the other four patients, detection of EDA in ctDNA preceded radiographic disease progression by 8, 14, 25, and 38 weeks, respectively (Fig. 3). Importantly, ctDNA‐based EDA was seen in three of those four patients coincident with radiographic evaluations that alone were interpreted by experienced radiologists as demonstrating no evidence of neoplastic disease. A representative example is shown in Fig. 4.

## 4 Discussion

Although several studies have shown how ctDNA data used in clinical oncology testing paradigms can reflect disease burden and responses to therapy, our investigation is among very few that provide direct evidence of its impact on clinical outcomes and interpretation of radiographic data (i.e., clinical utility) in patients with melanoma in a real‐world setting. First, the detection of ctDNA revealed targetable mutations where tumor testing did not, allowing for administration of and response to targeted therapy. Second, ctDNA revealed EDA in advance of radiographic progression, effectively shortening PFS.

Melanoma lends itself nicely to such a ‘liquid monitoring’ approach based on the presence of hotspot somatic mutations in a large percentage of tumors. In one study of 318 melanoma specimens, mutations in BRAF or NRAS were detected in 166 (52%) and 88 (28%), respectively (Cancer Genome Atlas Network, 2015). However, intra‐ and intertumor mutational heterogeneity is well‐described in melanoma and other cancers (Gerlinger *et al*., 2012; Yancovitz *et al*., 2012). This has important clinical implications for patients with advanced disease in need of therapeutic options, as an assessment of targetable mutations in a single tumor (even the primary lesion) may not provide a representative real‐time assessment of the patient's malignancy in a holistic manner. Indeed, of the 19 evaluable patients in our study whose tumor tissue analysis failed to reveal one of the seven aforementioned mutations in *BRAF* or *NRAS*, one patient (1/19, 5%) was found to have a circulating *BRAF* V600E mutation. As a result, this patient was treated with combination BRAF/MEK inhibitor therapy and experienced a partial response (RECIST 1.1). Assessing ctDNA may also sidestep the need for repeat tumor biopsies in patients whose tumor tissue quality or quantity is insufficient for genetic evaluation, or for patients with multiple primary lesions, each of which may have different targetable mutations (Adler *et al*., 2018). The noninvasive nature of ctDNA testing is particularly important for patients with metastases in locations that require invasive biopsy techniques. For one such patient in this study, ctDNA analysis revealed a targetable mutation, thereby obviating the need for repeat tumor biopsy and allowing for prompt treatment of this patient with targeted therapy, resulting in a partial response (RECIST 1.1). This study is, therefore, one of very few trials to prospectively assess treatment outcomes after administration of targeted therapy based exclusively on a ctDNA test result (Remon *et al*., 2017). Further investigation among larger groups of patients will be needed to demonstrate how frequently plasma‐based assessments of targetable somatic mutations improve patient outcomes when such a mutation is not revealed via tumor‐based molecular testing (Thierry *et al*., 2014; Wong *et al*., 2017).

Our study findings corroborate results from other trials demonstrating that ctDNA can provide evidence of tumor activity in advance of radiographic disease progression (Dawson *et al*., 2013; Diaz *et al*., 2012; Misale *et al*., 2012; Newman *et al*., 2014). Our observations also demonstrate that ctDNA interrogation can reveal EDA in patients who, after receiving therapy for advanced melanoma, appear to have no clinical or radiographic evidence of disease. Detection of disease below the resolution of current imaging modalities is particularly important in melanoma, which often metastasizes to unusual sites (Fig. 4; Fishman *et al*., 1990). The specificity (100%; 95% CI, 80.5–100) of the BEAMing assay used in this study also supports the use of ctDNA as a tumor marker alternative to serum lactate dehydrogenase (LDH), which has traditionally been used as a marker of melanoma activity. Unlike LDH, which has poor specificity (Egberts *et al*., 2009), detectable ctDNA specifically implies the presence of melanoma. The clinical utility of this finding is twofold. First, in this era of widely available standard‐of‐care and clinical trial‐based treatments for patients with advanced melanoma, timely blood‐based detection of disease progression could provide earlier opportunities for therapeutic intervention. For instance, in a recent pooled analysis of outcomes after treatment with dabrafenib and trametinib, patients with a smaller melanoma burden confined to < 3 organs comprised the most favorable prognostic group (Schadendorf *et al*., 2017). Based on findings from Gray and colleagues suggesting that lower pretreatment ctDNA levels are associated with prolonged PFS and response to targeted or immune‐based therapies, serial intrapatient ctDNA measurements may provide early opportunities for re‐initiation of therapy or transition to an alternative treatment regimen (Gray *et al*., 2015). From a research perspective, longitudinal intrapatient blood‐based detection of melanoma progression may have implications in the setting of translational/clinical trials in which surrogate endpoints such as clinical/radiographic progression‐free survival are used as markers of drug efficacy (Kemp and Prasad, 2017). Our findings indicate that duration of PFS measured using serial ctDNA levels may differ from results obtained radiographically. Larger studies, perhaps involving quantitative intrapatient measurements of ctDNA changes, will be required to further define appropriate surrogate endpoints. Second, in the setting of detectable ctDNA, contemporaneously performed clinical examinations and imaging studies should be interpreted with a high index of suspicion. An alternative imaging modality (e.g., positron emission tomography/CT scan) should be considered in patients with detectable ctDNA and no CT‐based evidence of melanoma (Forschner *et al*., 2017). Indeed, our investigation demonstrates that ctDNA measurements complement the current standard of care of serial clinical examinations and radiographic imaging.

Finally, serially undetectable ctDNA assessments appear to underscore radiographic and clinical evaluations in patients with durable responses to treatment. Although currently available targeted and immune‐based therapies have demonstrated remarkable antitumor efficacy in patients with advanced melanoma, such treatments are expensive and potentially toxic (Queirolo and Spagnolo, 2017; Teply and Lipson, 2014). Further studies, perhaps focused on particular therapies, are required to assess whether persistently undetectable ctDNA levels used as a complement to radiographic and clinical analyses might signal an appropriate time to discontinue particular drugs as clinicians aim to minimize cost while maximizing efficacy and safety of anti‐neoplastic treatment regimens.

The sensitivity of the BEAMing assay used in this study appears to be impacted both by disease burden and by location. Thus, it is not surprising that ctDNA levels did not become detectable in patients with only locoregionally recurrent disease in skin and lymph nodes (cohort B), whose tumors may not be spreading hematogenously. Among patients in cohort B who developed distant metastases, imaging and ctDNA identified progression at the same time point. In cases like these, ctDNA levels may aid in clarifying indeterminate imaging findings by influencing a radiologist's level of suspicion regarding abnormalities.

Similarly, we observed from the patients in cohort A that metastatic disease confined exclusively to the lungs and/or brain often failed to produce detectable amounts of ctDNA. These results mirror previous findings that ctDNA was frequently undetectable in the blood of patients with cancers that originate from or metastasize to the central nervous system (Bettegowda *et al*., 2014, De Mattos‐Arruda *et al*., 2015; Momtaz *et al*., 2016). While efforts are underway by several groups to improve the performance characteristics of various ctDNA detection methods, there may be some patients for whom ctDNA might not be a suitable biomarker based on tumor burden, location, or biology (Aravanis *et al*., 2017; Bettegowda *et al*., 2014, Shu *et al*., 2017). These patients require the continued use of surveillance imaging to assess disease status.

Our study results provide a basis for understanding the anatomical location of metastases and total tumor burden necessary for ctDNA to be reliably detectable among patients with advanced melanoma in a real‐world setting. Larger studies including patients with other cancer types will be required to better evaluate the suitability of plasma ctDNA testing on an individual patient basis.

Our findings provide a foundation for further evaluation of the utility of ctDNA in several settings. For example, although we considered ctDNA in binary terms (i.e., detectable vs undetectable), future investigations involving larger cohorts of patients might quantitate ctDNA as a marker of response to therapy in order to evaluate its utility in clinical research trials (i.e., as a complement to RECIST or immune RECIST criteria). Furthermore, ctDNA quantitation could be evaluated as a potential prognostic marker for incorporation into melanoma staging guidelines. Recent updates to the American Joint Committee on Cancer (AJCC) Eighth Edition Cancer Staging Manual continue to incorporate LDH, given its association with progression‐free and overall survival in recent studies of immune checkpoint blockers and oncogenic pathway inhibitors (Kelderman *et al*., 2014; Long *et al*., 2016; Nosrati *et al*., 2017).

## 5 Conclusions

In conclusion, our study results demonstrate that incorporating ctDNA assessments into real‐world melanoma patient management can influence patient care decisions, alter radiographic interpretations, and impact clinical outcomes. Our findings serve as a blueprint for future, randomized investigations designed to further evaluate the clinical utility of incorporating ctDNA among larger groups of patients with melanoma.

## Conflict of interest

PB received unrestricted educational grant from Bristol‐Myers Squibb. WHS received institutional research grants from Bristol‐Myers Squibb, Merck, Novartis, and consultant for Bristol‐Myers Squibb, Merck, Novartis, Castle Biosciences. EJL received institutional research grants from Bristol‐Myers Squibb and Merck, and consultant for Array Biopharma and Bristol‐Myers Squibb. HQ, DLE, and FSJ are employees of Sysmex Inostics, Inc. The other authors have no potential conflict of interests to declare.

## Author contributions

SPR and BL analyzed and interpreted data, and drafted and revised the manuscript. MM, PB, and JS acquired data and revised the manuscript. MDS acquired, analyzed, and interpreted data and edited the manuscript. HQ, DLE, FSJ, KBB, and WHS analyzed and interpreted data and revised the manuscript. EJL conceived of the project; acquired, analyzed, and interpreted data; and drafted and revised the manuscript. All authors read, critically revised, and approved the final manuscript.

## Supporting information


**Table S1.** ctDNA and radiographic outcomes data for all patients in Cohort B.
**Table S2.** ctDNA and radiographic outcomes data for all patients in Cohort C.
