# Supplementary material for: From validity to clinical utility: the influence of circulating tumor DNA on melanoma patient management in a real‐world setting
Source: Mol Oncol. 2018 Sep 8;12(10):1661–72. doi: 10.1002/1878-0261.12373 (PMC6165998; doi:10.1002/1878-0261.12373)
Supplement: Supplementary file 1 — Table S1. ctDNA and radiographic outcomes data for all patients in Cohort B. Table S2. ctDNA and radiographic outcomes data for all patients in Cohort C. [file MOL2-12-1661-s001.docx]

|  |  |  |  | **Time point 1** | | **Time point 2** | | **Time point 3** | | **Time point 4** | | **Time point 5** | |
| --- | --- | --- | --- | --- | --- | --- | --- | --- | --- | --- | --- | --- | --- |
| **Patient ID** | **Melanoma stage at enrollment (AJCC ver. 7)** | **Mutation** | | **ctDNA result, MAF (MM/ml)** | **radiographic findings** | **ctDNA result, MAF (MM/ml)** | **radiographic findings** | **ctDNA result, MAF (MM/ml)** | **radiographic findings** | **ctDNA result, MAF (MM/ml)** | **radiographic findings** | **ctDNA result, MAF (MM/ml)** | **radiographic findings** |
| 1 | IIIB | BRAF | V600E | ND | N.E.D. | 0.3879 (8.14) | liver, lung mets | 28.1149 (2,338) | tumor progression (poor adherence to targeted Rx) | 1.6527 (488.0) | tumor regression | 0.0855 (3.11) | tumor regression |
| 2 | IIIA | NRAS | Q61K | ND | N.E.D. | ND | NP | ND | N.E.D. | ND | N.E.D. |  |  |
| 3 | IIIB | BRAF | V600E | ND | N.E.D. | ND | N.E.D. | ND | N.E.D. | ND | N.E.D. |  |  |
| 4 | IIIA | BRAF | V600E | ND | N.E.D. | ND | N.E.D. | ND | N.E.D. | ND | N.E.D. |  |  |
| 5 | IIIA | NRAS | Q61K | ND | N.E.D. | ND | NP | ND | N.E.D. | ND | N.E.D. |  |  |
| 6 | IIIB | BRAF | V600E | ND | N.E.D. | ND | NP | ND | N.E.D. | ND | N.E.D. |  |  |
| 7 | IIIB | NRAS | Q61R | ND | N.E.D. | ND | N.E.D. | ND | N.E.D. | ND | N.E.D. |  |  |
| 8 | IIIC | BRAF | V600E | ND | N.E.D. | ND | NP | ND | N.E.D. | ND | N.E.D. |  |  |
| 9 | IIIC | NRAS | Q61R | ND | N.E.D. | ND | N.E.D. | ND | Local recurrence (1 cm LN) | ND | N.E.D. |  |  |
| 10 | IIIA | BRAF | V600E | ND | N.E.D. | ND | N.E.D. | ND | N.E.D. |  |  |  |  |
| 11 | IIIA | BRAF | V600E | ND | N.E.D. | ND | N.E.D. | ND | N.E.D. |  |  |  |  |
| 12 | IIIB | BRAF | V600E | ND | N.E.D. | ND | N.E.D. | ND | N.E.D. |  |  |  |  |
| 13 | IIIC | NRAS | Q61H (a183t) | ND | N.E.D. | ND | N.E.D. | ND | N.E.D. |  |  |  |  |
| 14 | IIIC | BRAF | V600E | ND | N.E.D. | ND | N.E.D. | ND | N.E.D. |  |  |  |  |
| 15 | IV | BRAF | V600E | ND | Local recurrence (L axillary LN met) | ND | lung met | ND | lung met resected; new 3mm subcutaneous mets |  |  |  |  |
| 16 | IIIC | NRAS | Q61K | ND | N.E.D. | ND | N.E.D. | ND | N.E.D. |  |  |  |  |
| 17 | IIIC | NRAS | Q61L | ND | N.E.D. | ND | N.E.D. |  |  |  |  |  |  |
| 18 | IIIB | BRAF | V600E | ND | N.E.D. | ND | N.E.D. |  |  |  |  |  |  |
| 19 | IIIC | BRAF | V600E | ND | N.E.D. | ND | N.E.D. |  |  |  |  |  |  |
| 20 | IIIB | BRAF | V600E | ND | N.E.D. | ND | N.E.D. |  |  |  |  |  |  |
| 21 | IIIA | BRAF | V600E | ND | N.E.D. | ND | N.E.D. |  |  |  |  |  |  |
| 22 | IIIC | BRAF | V600E | ND | N.E.D. | ND | N.E.D. |  |  |  |  |  |  |
| 23 | IIIB | BRAF | V600E | ND | N.E.D. | ND | N.E.D. |  |  |  |  |  |  |
| 24 | IIB | BRAF | V600K | ND | N.E.D. | ND | N.E.D. |  |  |  |  |  |  |
| 25 | IIIA | BRAF | V600E | ND | N.E.D. | ND | N.E.D. |  |  |  |  |  |  |
| 26 | IIIA | NRAS | Q61R | 1.474 (6.13) | 2cm kidney met |  |  |  |  |  |  |  |  |
| 27 | IIIC | BRAF | V600K | ND | Local recurrence (1.6cm subcutaneous met) |  |  |  |  |  |  |  |  |
| 28 | IIIC | NRAS | Q61K | ND | N.E.D. |  |  |  |  |  |  |  |  |
| 29 | IIIB | NRAS | Q61L | ND | N.E.D. |  |  |  |  |  |  |  |  |

**Supplementary Table S1: ctDNA and radiographic outcomes data for all patients in Cohort B.** Yellow highlights indicate plasma specimens in which ctDNA was detected. Red highlights indicate locoregional melanoma recurrence. Green highlights indicate radiographic appearance of a distant melanoma metastasis. (LN, lymph node; MAF, mutant allelic fraction; MM/ml, mutant molecules per milliliter plasma; ND, not detected; NP, not performed)

|  | **Patient ID** | **Tumor mutation (tissue-based)** | | **Therapy** | **Timepoint 1** | | **Timepoint 2** | | **Timepoint 3** | | **Timepoint 4** | | **Timepoint 5** | | **Timepoint 6** | |
| --- | --- | --- | --- | --- | --- | --- | --- | --- | --- | --- | --- | --- | --- | --- | --- | --- |
|  |  |  |  |  | **ctDNA result, MAF (MM/ml)** | **radiographic findings** | **ctDNA result, MAF (MM/ml)** | **radiographic findings** | **ctDNA result, MAF (MM/ml)** | **radiographic findings** | **ctDNA result, MAF (MM/ml)** | **radiographic findings** | **ctDNA result, MAF (MM/ml)** | **radiographic findings** | **ctDNA result, MAF (MM/ml)** | **radiographic findings** |
| Partial or complete response (PR, CR); no EDA in ctDNA after baseline | 1 | BRAF | V600E | anti-PD-1 | ND | prior PR | ND | PR | ND | PR | ND | PR | ND | PR |  |  |
|  | 2 | NRAS | Q61K | IL-2, metastatectomy | ND | prior N.E.D. | ND | N.E.D. | ND | N.E.D. | ND | N.E.D |  |  |  |  |
|  | 3 | BRAF | V600E | anti-CTLA-4+anti-PD-1 | ND | prior CR | ND | CR | ND | CR | N/A | CR |  |  |  |  |
|  | 4 | NRAS | Q61R | anti-CTLA-4 + RT | ND | prior PR | ND | PR | ND | PR | N/A | PR |  |  |  |  |
|  | 5 | BRAF | V600E | anti-CTLA-4+anti-PD-1 | ND | prior CR | ND | CR | ND | CR | N/A | CR |  |  |  |  |
|  | 6 | BRAF | V600E | anti-PD-1 | ND | prior CR | ND | CR | ND | CR | N/A | CR |  |  |  |  |
|  | 7 | NRAS | Q61R | anti-PD-1 | ND | prior CR | ND | CR | ND | CR | N/A | PR |  |  |  |  |
|  | 8 | BRAF | V600E | anti-PD-1 | ND | prior PR | ND | PR | ND | PR | ND | PR |  |  |  |  |
|  | 9 | BRAF | V600E | anti-CTLA-4+anti-PD-1 | ND | prior PR | ND | PR | ND | PR | ND | PR |  |  |  |  |
|  | 10 | NRAS | Q61R | combination ICI | 0.2936 (2.96) | baseline | ND | PR | ND | PR | ND | PR |  |  |  |  |
|  | 11 | BRAF | V600E | anti-CTLA-4+anti-PD-1 | ND | prior PR | ND | PR | ND | PR |  |  |  |  |  |  |
|  | 12 | BRAF | V600E | BRAF+MEK | ND | prior PR | ND | PR | N/A | PR |  |  |  |  |  |  |
|  | 13 | NRAS | Q61R | anti-PD-1 | ND | prior PR | ND | PR | ND | PR |  |  |  |  |  |  |
|  | 14 | BRAF | V600E | anti-CTLA-4+anti-PD-1 | ND | prior PR | ND | PR | ND | PR |  |  |  |  |  |  |
|  | 15 | BRAF | V600E | temozolomide + RT | ND | prior PR | ND | PR |  |  |  |  |  |  |  |  |
|  | 16 | NRAS | Q61R | combination ICI | ND | prior PR | ND | PR |  |  |  |  |  |  |  |  |
|  | 17 | BRAF | V600E | anti-CTLA-4+anti-PD-1 + RT | ND | prior PR | ND | PR |  |  |  |  |  |  |  |  |
| PR, then PD after end-of-study | 18 | NRAS | Q61R | anti-CTLA-4+anti-PD-1 | 0.2618 (7.86) | prior SD | 0.4206 (9.96) | PR | 4.6949 (98.61) | PR | 0.5276 (10.3) | PR |  |  |  |  |
| PR, Rx change due to toxicity | 19 | BRAF | V600K | BRAF+MEK inhibitors, then anti-CTLA-4+anti-PD-1 | 25.4087 (18,236.94) | baseline | 23.2646 (6.93) | PR | 62.9906 (33.02) | baseline for new therapy |  |  |  |  |  |  |
| Stable disease | 20 | BRAF | V600E | anti-PD-1 | 22.5854 (1788.01) | baseline | 20.5157 (1014.36) | SD | 10.6381 (393.92) | SD |  |  |  |  |  |  |
|  | 21 | BRAF | V600E | anti-PD-1 + RT | 0.2603 (7.43) | baseline | 0.1675 (3.52) | SD | 0.1587 (2.195) | SD |  |  |  |  |  |  |
|  | 22 | BRAF | V600E | BRAF+MEK inhibitors | 0.7049 (31.09) | baseline | ND | SD |  |  |  |  |  |  |  |  |
| Progressive disease | 23 | NRAS | Q61R | anti-CTLA-4+anti-PD-1 | 5.1307 (100.24) | baseline | ND | PR | 0.0486 (0.54) | CR | ND | CR | 0.0756 (1.35) | PD | 0.0503 (0.70) | baseline for re-induction anti-PD-1 |
|  | 24 | BRAF | V600E | anti-PD-1 | 0.9461 (53.41) | prior CR | NP | CR | 5.7012 (256.42) | PD | 6.62 (627.36) | PD | 1.05 (108.64) | PD | 30.23 (11389.20) | PD |
|  | 25 | BRAF | V600E | anti-PD-1 | 0.0306 (0.68) | baseline | ND | PR | 0.1267 (2.5) | PD | ND | PR to 2nd line Rx | ND | PR to 2nd line Rx |  |  |
|  | 26 | BRAF | V600E | metastatectomy, anti-CTLA-4 | ND | prior N.E.D. | 0.5965 (21.4) | N.E.D | 1.2986 (31.43) | N.E.D | 3.5429 (68.12) | N.E.D | 7.8046 (150.87) | PD |  |  |
|  | 27 | BRAF | V600K | BRAF+MEK inhibitors | ND | prior Non-CR/Non-PD | 0.1982 (3.38) | Non-CR/Non-PD | 0.6791 (7.61) | PD | N/A | PD | N/A | PD |  |  |
|  | 28 | BRAF | V600E | anti-PD-1 | ND | baseline | 0.1044 (5.56) | PD | 0.1243 (4.88) | PD |  |  |  |  |  |  |
|  | 29 | BRAF | V600E | combination ICI | 0.124 (5.7) | baseline | 0.0452 (0.89) | PD | 3.784 (85.13) | PD |  |  |  |  |  |  |
|  | 30 | NRAS | Q61R | MEK inhibitor + RT | 1.1915 (44.22) | baseline | 19.6485 (2685.55) | PD |  |  |  |  |  |  |  |  |

**Supplementary Table S2: ctDNA and radiographic outcomes data for all patients in Cohort C.** Green highlights indicate plasma specimens in which ctDNA was detected. Red highlights indicate first occurrence of radiographically-detected progressive melanoma (RECIST 1.1). (ctDNA, circulating tumor DNA; CR, complete response; EDA, evidence of disease activity in ctDNA; ICI, immune checkpoint inhibitors; MAF, mutant allelic fraction; MM/ml, mutant molecules per milliliter plasma; ND, not detected; N.E.D., no radiographic evidence of disease; NP, not performed; PD, progressive disease; PR, partial response; RT, radiation therapy; Rx, therapy)
